# New Avenues Towards Prevention and Treatment of Mucositis Induced by Cancer Therapies

**DOI:** 10.1177/15347354211012753

**Published:** 2021-05-08

**Authors:** Angela R. Zambrano, Francisco J. Bonilla-Escobar

**Affiliations:** 1 Universidad Icesi, Faculty of Health Sciences. Centro de Investigaciones Clínicas, Fundación Valle del Lili, Cali, Valle del Cauca, Colombia; 2 Somos Ciencia al Servicio de la Comunidad, Fundación SCISCO / Science to Serve the Community, SCISCO Foundation, Cali, Colombia; 3 Universidad del Valle, Cali, Colombia; 4 University of Pittsburgh, PA, USA

Mucositis is a prevalent issue in patients under cancer treatment which can cause delay in the therapy and therefore potentially affects outcomes and survival. Several therapeutic options have been studied to prevent or treat mucositis^1,2^; however, the lack of standardization of the therapies as well as their diversity and limitations in the methods in which they are assessed,^2^ has left patients and healthcare providers without the proper tools to battle this common enemy.

The article by Steinmann et al^1^ highlights a trend that has become relevant in recent years in the management of mucositis: naturopathic and anthroposophic medicine. This study is relevant given that alternatives to the “magic mouthwash” are needed and strategies towards standardization of mouthwashes preparation are required.^3^ As per the findings of the authors and the study limitations, most of the interventions that were described as efficient by the experts lack a process of evaluation based on research and came from single centers.^1^ On the other hand, the description of the preparation of rinses did not guarantee that the mechanism of action behind the used substance was kept. Knowing for example, that the antioxidant properties of the teas remained after the preparation is relevant to guarantee an impact in the chain of inflammation and cellular damage caused by the cancer therapy.

One of the key points about this study is the need for stronger research on alternative therapies for mucositis induced by cancer treatment. Future research not only needs stronger designs but multidisciplinary approaches. Building evidence about interventions can prevent us from falling into individualistic fallacies and improve what we do for patients worldwide. Another recent systematic review found that lack of randomization, lack of standardization, small sample sizes, and baseline differences were only a few of the items to address in future research in mucositis.^2^ A deeper understanding of herbal products is required to guarantee that the healing properties of these substances are kept even after a preparation process.

It calls our attention that one of the evaluated formulas included lidocaine in combination with extracts of chamomile flowers. While lidocaine serves to treat pain, it is expected that chamomile action is based on its antioxidant properties. However, there is stronger evidence around the safety and efficacy of morphine in pain management in mucositis,^4^ as well as other promising antioxidants from the family of teas including calendula and green tea.^5^ Research on this kind of combinations, using herbal extracts and well-understood drugs could lead to better patient-centered outcomes.

There is still a long pathway towards strengthening the evidence around prevention and treatment of mucositis, but efforts like the one described by Steinmann et al, showing that alternative therapies are being used with some degree of evidence, are vital to promoting more and robust research in the field.
